# Associated Factors of Fall Risk in Older Adults Living in the Community: Insights from the FRRISque Assessment

**DOI:** 10.1093/geroni/igaf122.3710

**Published:** 2025-12-31

**Authors:** Alvaro Maciel, Sabrina Macedo, Paulo Barbosa, Wesley Sales, Edgar Vieira

**Affiliations:** Universidade Federal do Rio Grande do Norte, NATAL, Rio Grande do Norte, Brazil; Universidade Federal do Rio Grande do Norte, Universidade Federal do Rio Grande do Norte, Rio Grande do Norte, Brazil; universidade Estadual da Paraiba, NATAL, Rio Grande do Norte, Brazil; Universidade Federal do Rio Grande do Norte, NATAL, Rio Grande do Norte, Brazil; FLORIDA INTERNATIONAL UNIVERSITY, MIAMI, Florida, United States

## Abstract

Aging reduces physiological reserves and mobility, increasing vulnerability to falls, which are a major public health concern in older populations. Identifying predictive factors for fall risk is essential for implementing effective prevention strategies. This cross-sectional study, part of the PRO-EVA project, included community-dwelling adults aged 60 years or older residing in Parnamirim, Brazil. Sociodemographic characteristics, lifestyle habits, health conditions, and measures of physical performance and cognitive function were collected using the Older Adults’ Health Record Booklet. Fall risk was assessed with the FRRISque questionnaire score. Linear regression analysis was performed to identify associations between potential predictors and fall risk. The study was approved by the Research Ethics Committee of the Federal University of Rio Grande do Norte (protocol no. 2.996.329). The sample comprised 735 participants, with a mean age of 70.1 years (±7.1), predominantly female (60.8%). Regression analysis revealed significant predictors of fall risk: sex (p = 0.03), polypharmacy defined as the use of five or more medications (p = 0.001), total Short Physical Performance Battery (SPPB) score (p = 0.01), presence of comorbidities (p = 0.05), and pain (p = 0.01). The adjusted model explained 25% of the variance in fall risk (R² = 0.25). These findings underscore the relevance of clinical and functional factors, such as sex, polypharmacy, physical performance, comorbidities, and pain, in predicting fall risk among older adults. Monitoring these predictors may guide targeted interventions to enhance safety and quality of life in this population.

